# Complete genome sequences of *Staphylococcus aureus* isolates with a novel sequence type from a patient with hospital-acquired pneumonia

**DOI:** 10.1128/mra.01282-25

**Published:** 2026-01-28

**Authors:** Daniel B. Amusin, Sophia H. Nozick, Richard G. Wunderink, Egon A. Ozer, Alan R. Hauser

**Affiliations:** 1 Department of Microbiology-Immunology, Northwestern University Feinberg School of Medicine 12244, Chicago, Illinois, USA; 2 Department of Medicine, Division of Infectious Diseases, Northwestern University Feinberg School of Medicine 12244, Chicago, Illinois, USA; 3 Department of Medicine, Division of Pulmonary and Critical Care, Northwestern University Feinberg School of Medicine 12244, Chicago, Illinois, USA; University of Maryland, Baltimore, Maryland, USA

**Keywords:** *Staphylococcus aureus*, sequence type, pneumonia

## Abstract

The complete genome of a *Staphylococcus aureus* isolate causing hospital-acquired pneumonia was sequenced. Genetic changes in subsequent isolates (cultured 8, 20, and 22 days later) from the lungs of the patient were identified. The genome comprised a 2.74 Mb chromosome encoding 2,658 genes, representing a novel sequence type designated ST10248.

## ANNOUNCEMENT

*Staphylococcus aureus* commonly causes hospital-acquired pneumonia (1). We utilized whole-genome sequencing to investigate the genomes of methicillin-susceptible *S. aureus* isolated from a mechanically ventilated patient at Northwestern Memorial Hospital in Chicago, USA, with hospital-acquired pneumonia (Northwestern University IRB #STU00204868).

Bronchoalveolar lavage fluids were collected by bronchoscopy at days 0 (SCRIPT-46514), 8 (SCRIPT-59750), 20 (SCRIPT-63970), and 22 (SCRIPT-68196) of the pneumonia episode. Fluids were cultured on blood agar (all cultures 16–20 h, 37°C). Single colonies were sub-cultured and speciated by MALDI-TOF. Isolates were sub-cultured on Luria-Bertani agar followed by broth and archived in 25% glycerol at −80°C. For short-read sequencing, stocks were cultured on Luria-Bertani agar, and single colonies were incubated in Luria-Bertani broth. DNA was extracted with the Promega Maxwell 16 system from culture incubated in lysostaphin solution (200 µg/mL lysostaphin, 20 mM Tris-HCl, 10 mM EDTA) for ~1 h. Sequencing libraries were constructed from unsheared DNA with an Illumina Nextera Kit and sequenced by Illumina NextSeq 500 to generate 150 bp paired-end reads. Nanopore sequencing was performed on SCRIPT-46514 from cultures grown on tryptic soy agar/broth, DNA was extracted with the Qiagen EZ2 Connect Tissue Kit, and the sequencing library was prepared from unsheared DNA with the Oxford Nanopore Native Barcoding Kit 24, using the included Long Fragment Buffer to enrich fragments >3 kb. Sequencing was performed on a GridION sequencer with an R10.4.1 flow cell for 72 h and basecalled with Dorado v7.6.7 using the super-accurate model (v4.3.0, 400 bp) with a minimum quality score of 10 (https://github.com/nanoporetech/dorado).

The complete sequence of SCRIPT-46514 was generated by assigning reads to eight subsamples, each assembled by Flye v2.9.5/Raven v1.8.3/miniasm v0.3 (polished by minipolish v1.2) (2–5). These were reconciled by AutoCycler v0.2.1 (6). All bioinformatic analyses used default parameters unless otherwise specified. The assembly was reoriented to begin with *dnaA* using dnaapler v0.8.1 and polished using medaka v2.0.1 (Nanopore reads) and polypolish v0.6.0 and pypolca v0.3.1 (Illumina reads) (7–9). The assembled genome comprised a circular 2,740,068 bp chromosome (GC: 32.87%) and was annotated by PGAP v6.9 to contain 2,658 genes (10). Multi-locus sequence typing (MLST) identified a novel *yqiL* allele, which was deposited into PubMLST; the isolate was subsequently assigned ST10248 (11).

Short-read sequencing was performed for the longitudinal isolates SCRIPT-59750, SCRIPT-63970, and SCRIPT-68196. Reads were trimmed with fastp v0.24.0 and assembled using SPAdes v3.9.1 with “automatic coverage cutoff” and “careful” parameters (12, 13). Contigs shorter than 200 bp or with average coverage <5 were removed. These assemblies shared the MLST of SCRIPT-46514. Single-nucleotide variants (SNVs) relative to the first isolate were called by alignment with BWA-MEM v0.7.19, Freebayes v1.3.9 (filtered to quality score > 20, depth > 10), and snpEff v5.2 (14–16). Assemblies from longitudinal isolates contained 32 contigs with lengths ranging from 2.71 to 2.75 Mb (Table 1). The isolates from days 20 and 22 carried four total SNVs, as shown in Table 1. Differences in assembly length were investigated by nucmer alignment to SCRIPT-46514 using MUMmer v3.23 (17). SCRIPT-63970 and SCRIPT-68196 both carried an unaligned 34.3 kb contig, which mapped to plasmid p0915 (GenBank CP148082.1) by BLAST (18). These data demonstrate plasticity of the *S. aureus* genome during acute infection and warrant further study.

**TABLE 1:** Genome assembly information^*a*^

	SCRIPT-46514	SCRIPT-59750	SCRIPT-63970	SCRIPT-68196
Pneumonia episode day	0	8	20	22
Nanopore reads (N50)	2,661,868 (2,695)	–	–	–
Illumina read pairs	1,495,489	2,363,584	2,492,306	2,388,862
Total assembly length	2,740,068	2,712,332	2,745,332	2,745,779
Assembly contigs	1	32	32	32
Assembly %GC	32.87	32.72	32.67	32.67
Assembly N50	–	325,930	325,591	325,699
SNVs relative to day 0	–	None	*qoxB* (985,674; A→G) BCCT family transporter (1,294,816; G→A) Intergenic region (1,809,881; A→G) *kdpA* (2,068,493; A→G)	*rpoC* (534,357; A→T) Intergenic region (600,240; C→A) *qoxB* (985,674; A→G) *kdpA* (2,068,493; A→G)

^*a*^ –, not applicable or not performed.

## Data Availability

The novel *yqiL* allele and multi-locus sequence type allele profile were deposited to PubMLST.org and assigned IDs 1313 and 10248, respectively. Whole-genome sequencing reads and assemblies were deposited to NCBI under the following BioSample, Sequence Read Archive, and GenBank accession numbers: SCRIPT-46514 (day 0; SAMN50926487; short reads SRR35228309; long reads SRR35228308; CP199996), SCRIPT-59750 (day 8; SAMN50926508; SRR35228307; JBQWBI000000000), SCRIPT-63970 (day 20; SAMN50926509; SRR35228306; JBQWBH000000000), and SCRIPT-68196 (day 22; SAMN50926510; SRR35228305; JBQWBG000000000).

## References

[1] Jones RN. 2010. Microbial etiologies of hospital-acquired bacterial pneumonia and ventilator-associated bacterial pneumonia. Clin Infect Dis 51:S81–S87. doi:10.1086/653053 20597676

[2] Kolmogorov M, Yuan J, Lin Y, Pevzner PA. 2019. Assembly of long, error-prone reads using repeat graphs. Nat Biotechnol 37:540–546. doi:10.1038/s41587-019-0072-8 30936562

[3] Vaser R, Šikić M. 2021. Time- and memory-efficient genome assembly with Raven. Nat Comput Sci 1:332–336. doi:10.1038/s43588-021-00073-4 38217213

[4] Li H. 2016. Minimap and miniasm: fast mapping and de novo assembly for noisy long sequences. Bioinformatics 32:2103–2110. doi:10.1093/bioinformatics/btw152 27153593 PMC4937194

[5] Wick RR, Holt KE. 2019. Benchmarking of long-read assemblers for prokaryote whole genome sequencing. F1000Res 8:2138. doi:10.12688/f1000research.21782.4 31984131 PMC6966772

[6] Wick RR, Howden BP, Stinear TP. 2025. Autocycler: long-read consensus assembly for bacterial genomes. Bioinformatics 41:btaf474. doi:10.1093/bioinformatics/btaf474 40875535 PMC12460055

[7] Bouras G, Grigson SR, Papudeshi B, Mallawaarachchi V, Roach MJ. 2024. Dnaapler: a tool to reorient circular microbial genomes. JOSS 9:5968. doi:10.21105/joss.05968

[8] Bouras G, Judd LM, Edwards RA, Vreugde S, Stinear TP, Wick RR. 2024. How low can you go? Short-read polishing of Oxford Nanopore bacterial genome assemblies. Microb Genom 10:001254. doi:10.1099/mgen.0.001254 38833287 PMC11261834

[9] Wick RR, Holt KE. 2022. Polypolish: short-read polishing of long-read bacterial genome assemblies. PLoS Comput Biol 18:e1009802. doi:10.1371/journal.pcbi.1009802 35073327 PMC8812927

[10] Li W, O’Neill KR, Haft DH, DiCuccio M, Chetvernin V, Badretdin A, Coulouris G, Chitsaz F, Derbyshire MK, Durkin AS, Gonzales NR, Gwadz M, Lanczycki CJ, Song JS, Thanki N, Wang J, Yamashita RA, Yang M, Zheng C, Marchler-Bauer A, Thibaud-Nissen F. 2021. RefSeq: expanding the prokaryotic genome annotation pipeline reach with protein family model curation. Nucleic Acids Res 49:D1020–D1028. doi:10.1093/nar/gkaa1105 33270901 PMC7779008

[11] Jolley KA, Bray JE, Maiden MCJ. 2018. Open-access bacterial population genomics: BIGSdb software, the PubMLST.org website and their applications. Wellcome Open Res 3:124. doi:10.12688/wellcomeopenres.14826.1 30345391 PMC6192448

[12] Prjibelski A, Antipov D, Meleshko D, Lapidus A, Korobeynikov A. 2020. Using SPAdes de novo assembler. CP in Bioinformatics 70:e102. doi:10.1002/cpbi.102 32559359

[13] Chen S, Zhou Y, Chen Y, Gu J. 2018. fastp: an ultra-fast all-in-one FASTQ preprocessor. Bioinformatics 34:i884–i890. doi:10.1093/bioinformatics/bty560 30423086 PMC6129281

[14] Li H. 2013. Aligning sequence reads, clone sequences and assembly contigs with BWA-MEM. arXiv. doi:10.48550/arXiv.1303.3997

[15] Garrison E, Marth G. 2012. Haplotype-based variant detection from short-read sequencing. arXiv. doi:10.48550/arXiv.1207.3907

[16] Cingolani P, Platts A, Wang LL, Coon M, Nguyen T, Wang L, Land SJ, Lu X, Ruden DM. 2012. A program for annotating and predicting the effects of single nucleotide polymorphisms, SnpEff: SNPs in the genome of Drosophila melanogaster strain w1118; iso-2; iso-3. Fly (Austin) 6:80–92. doi:10.4161/fly.19695 22728672 PMC3679285

[17] Kurtz S, Phillippy A, Delcher AL, Smoot M, Shumway M, Antonescu C, Salzberg SL. 2004. Versatile and open software for comparing large genomes. Genome Biol 5:R12. doi:10.1186/gb-2004-5-2-r12 14759262 PMC395750

[18] Zhang Z, Schwartz S, Wagner L, Miller W. 2000. A greedy algorithm for aligning DNA sequences. J Comput Biol 7:203–214. doi:10.1089/10665270050081478 10890397

